# Supplementary figures and images for: Mast cell specific Cyp11a1 deficiency promotes T cell mediated immunity and suppresses tumour metastasis in a mouse model of melanoma
Source: Sci Rep. 2026 Jun 2;16:16983. doi: 10.1038/s41598-026-56344-5 (PMC13230768; doi:10.1038/s41598-026-56344-5)

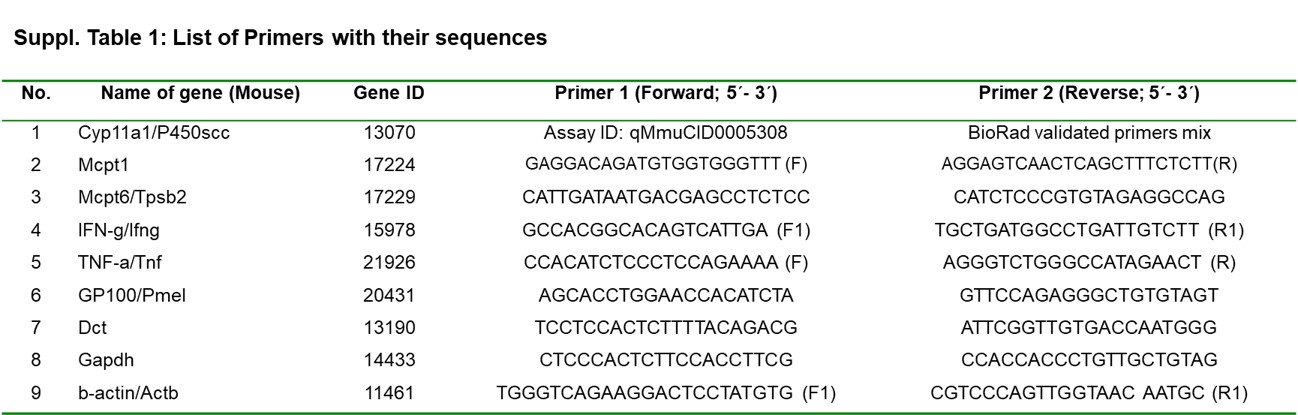

Supplement: Supplementary file 1 — Supplementary Material 1 [file 41598_2026_56344_MOESM1_ESM.zip › Supplementary Table 1.jpg]

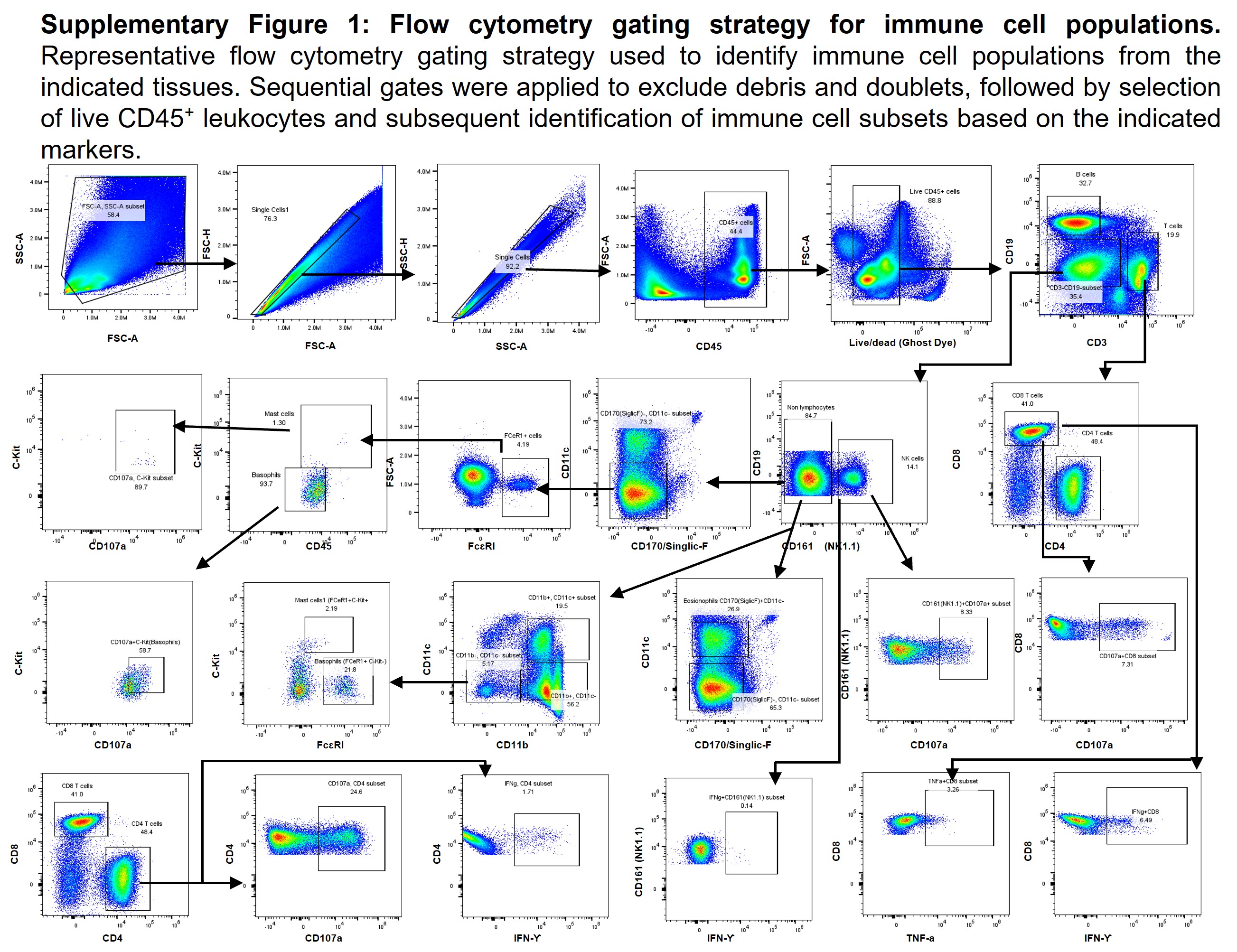

Supplement: Supplementary file 2 — Supplementary Material 2 [file 41598_2026_56344_MOESM2_ESM.zip › Supplementary Figure 1.jpg]
